# Complete Genome Sequence of *Clostridium stercorarium* subsp. *stercorarium* Strain DSM 8532, a Thermophilic Degrader of Plant Cell Wall Fibers

**DOI:** 10.1128/genomeA.00073-13

**Published:** 2013-03-07

**Authors:** Anja Poehlein, Vladimir V. Zverlov, Rolf Daniel, Wolfgang H. Schwarz, Wolfgang Liebl

**Affiliations:** a Georg-August-University Goettingen, Institute of Microbiology and Genetics, Department of Genomic and Applied Microbiology & Goettingen Genomics Laboratory, Göttingen, Germany; b Technische Universität München, Department of Microbiology, Freising-Weihenstephan, Germany; c Institute of Molecular Genetics, Russian Academy of Science, Moscow, Russian Federation

## Abstract

*Clostridium stercorarium* strain DSM 8532 is a thermophilic bacterium capable of efficiently degrading polysaccharides in plant biomass and converting the resulting sugars to ethanol and acetate. The complete genome sequence of 2.96 Mbp reveals a multitude of genes for hydrolytic enzymes and enables further study of the organism and its enzymes, and their exploitation for biotechnological processes.

## GENOME ANNOUNCEMENT

*Clostridium stercorarium* is a ubiquitous, thermophilic bacterial species. It degrades polysaccharides in plant biomass and produces acetate, ethanol, CO_2_, and H_2_, as well as minor amounts of lactate and l-alanine (1, 2, 3, 22). The three species *C. stercorarium, Thermobacteroides leptospartum*, and *C. thermolacticum* have been unified to the species *C. stercorarium*, with strain *C. stercorarium* subsp. *stercorarium* DSM 8532 as the type strain, and this species was recently regrouped to the family *Ruminococcaceae* within the order *Clostridiales* (1, 4, 5).

The two-component cellulase system of *C. stercorarium* is regarded as a model for the most simple cellulase system able to degrade crystalline cellulose (6). *C. stercorarium* has been detected in thermophilic biogas plants, in which it plays a major role in plant biomass degradation (7). A great number of hemicellulases, glycosidases, and esterases are produced by *C. stercorarium* and have been investigated and cloned (8, 9, 10, 11, 12, 13). The α-rhamnosidase RamA was applied for debittering of citrus juices (14).

*C. stercorarium* is especially suited for the fermentation of hemicellulose to organic solvents. Isolates have been used in Japan in a single-step ethanol-fermenting pilot process with lignocellulosic biomass as the substrate (15).

The genome sequencing of *C. stercorarium* was carried out with a combined approach using the 454 GS-FLX system (titanium GS70 chemistry; Roche Life Science, Mannheim, Germany) and the Genome Analyzer II (Illumina, San Diego, CA), resulting in average coverage of 14.69 and 66.88, respectively. The initial assembly, performed by employing the MIRA software (16), yielded 60 remaining gaps, which were closed with PCR-based techniques and Sanger sequencing of the products. The final sequence of *C. stercorarium* subsp. *stercorarium* DSM 8532 comprises one circular chromosome with a size of 2.96 Mb and an overall G+C content of 42.25 mol%. The functional annotation of the 2,687 protein-coding genes was initially carried out with the IMG/ER (Intergrated Microbial Genomes/Expert Review) system (17) and manually curated by using the Swiss-Prot, TREMBL, and InterPro databases (18). The genome harbors 3 rRNA operons and 48 tRNA genes, which were identified with RNAmmer and tRNAscan, respectively (19, 20). The genes for tRNA^Sec^ and for selenocysteine incorporation are missing. We identified 10 CRISPR loci with 3 to 63 repeats and 3 gene clusters encoding Cas proteins, 2 of which contain *cas8a1* and are classified as subtype I-A/Apern type (21). Approximately 82% of the 2,687 protein-coding genes (CDS) could be assigned to functions, and the remaining 474 CDS (18%) are hypothetical proteins (455 CDS) and pseudogenes (18 CDS). Blast analysis revealed that approximately 79% of the CDS could be allocated to the 21 functional clusters of orthologous groups (COGs), a percentage that is in the same range as described for other clostridia. The most abundant groups are replication, recombination, and repair (6.85%); amino acid transport and metabolism (8.13%); and carbohydrate transport and metabolism (12.13%). Compared to results for other clostridia, the number of CDS belonging to the last group is relatively high.

### Nucleotide sequence accession number.

The genome sequence of *Clostridium stercorarium* subsp. *stercorarium* DSM 8532 has been deposited in GenBank under accession number CP004044.
